# IL-1α protects intestinal barrier in murine visceral leishmaniasis

**DOI:** 10.1128/mbio.00657-26

**Published:** 2026-09-14

**Authors:** Laís A. Sacramento, Pedro Alexandre Sampaio, Gabriela Castilho Martins, Luciana Benevides, Claudia Gonzalez-Lombana, Gabrielle B. Trindade, Michely Mello, Fernando Q. Cunha, Livia S. Zaramela, João S. Silva, Phillip Scott, Vanessa Carregaro

**Affiliations:** 1 Department of Biochemistry and Immunology, Ribeirão Preto Medical School, University of São Paulo 54539, Ribeirão Preto, Brazil; 2 Department of Pathobiology, School of Veterinary Medicine, University of Pennsylvania 634332, Philadelphia, Pennsylvania, USA; 3 Academic Institute of Health and Biological Sciences, Goiás State University 271384 , Itumbiara, Brazil; 4 Fiocruz-Bi-Institutional Translational Medicine Project, Ribeirão Preto Medical School, University of São Paulo 54539, Ribeirão Preto, Brazil; 5 Department of Pharmacology, Ribeirão Preto Medical School, University of São Paulo 737194, Ribeirão Preto, Brazil; University of Virginia, Charlottesville, Virginia, USA

**Keywords:** interleukin-1, IFNγ-mediated response, intestinal integrity, visceral leishmaniasis

## Abstract

Interleukin-1 (IL-1) is a pleiotropic cytokine important in inflammatory and infectious diseases, but its roles in visceral leishmaniasis (VL) remain poorly understood. Here, we evaluated the role of IL-1 cytokines during experimental visceral leishmaniasis. We showed that IL-1 receptor 1 (IL-1R1) deficiency enhanced the gamma interferon (IFNγ)-mediated response and restricted parasites in an IL-1α-dependent manner, whereas IL-1β deficiency had no effect. The absence of IL-1R1 and IL-1α led to intestinal microbiota dysbiosis. The infection induced IL-1α expression in the gut. The absence of IL-1α led to reduced expression of tight-junction molecules, increased intestinal permeability, and bacterial translocation. These alterations amplified non-*Leishmania*-specific IFN-γ production, which was reversed by broad-spectrum antibiotic treatment. Bone marrow chimeric transplantation showed that hematopoietic cells are the source of IL-1α during VL. Together, our findings reveal that IL-1α acts as a key regulator of gut barrier integrity in VL and indirectly restrains systemic IFNγ-driven inflammation. This work highlights IL-1α-dependent intestinal protection as an overlooked component of VL pathogenesis.

**IMPORTANCE:**

Visceral leishmaniasis (VL) is a systemic and chronic disease caused by *Leishmania* parasites. Most infected individuals remain asymptomatic due to an appropriate IFNγ-mediated response. By contrast, the disease progression is characterized by excessive inflammation, with high levels of cytokines, among them IFNγ. IL-1 cytokines play a role in modulating immune responses and epithelial barriers. We report that IL-1α protects the intestinal environment and indirectly regulates the IFNγ-mediated response during experimental VL. The infection induces IL-1α expression in the intestine, and its absence leads to dysbiosis, gut permeability, and bacterial translocation. Overall, this study reveals the role of IL-1α in intestinal integrity during VL and in regulating the IFNγ-mediated response. Expanding the knowledge of IL-1α in intestinal protection may support therapeutic approaches to improve the outcomes of infection.

## INTRODUCTION

The interleukin-1 (IL-1) family of cytokines comprises 11 members with pleiotropic functions in inflammation and host defense (1). Among its members, IL-1α and IL-1β were the first to be described (2). Although both cytokines bind to the same IL-1 receptor (IL-1R1), several reports have demonstrated that IL-1α and IL-1β may display divergent activities (3). The functional differences exerted by IL-1α and IL-1β are related to their maturation processes and bioavailability (4). While IL-1β is produced by hematopoietic cells upon an inflammatory stimulus and requires inflammasome-dependent processing (5, 6), IL-1α is constitutively expressed and can be induced in hematopoietic cells and nonhematopoietic compartments by a wide range of stimuli, including microbial or inflammatory molecules (7, 8). IL-1α may act as an alarmin, initiating local inflammation as its bioactivity is independent of proteolytic processing and its bioactive form is induced by a wide range of insults, such as cell death due to injury or infectious disease (9).

IL-1 cytokines are central mediators of immunity in infectious diseases caused by viruses, bacteria, fungi, and parasites (10–13). Following their induction, IL-1α and IL-1β orchestrate immune mechanisms (14) and are also reported to act in the epithelial protection of the skin, lung, and intestine (15–17). IL-1-mediated responses have been widely studied in murine models of parasitic infections. Their functions in parasitic infections have been extensively examined in cutaneous leishmaniasis (CL), where IL-1 cytokines shape Th1 differentiation (18), macrophage activation (19), and immunopathology in some contexts (20–22). In contrast, the role of IL-1 signaling in visceral leishmaniasis (VL) remains poorly defined despite evidence of reduced IL-1 production in acute human disease and recovery after treatment (23) and that IL-1α promotes granuloma formation without impact on parasite control (24).

Visceral leishmaniasis (VL) is a systemic and chronic disease caused by *Leishmania* parasites. Infected individuals may exhibit subclinical symptoms or progress to clinical disease that affects the spleen, liver, and bone marrow. Intestinal involvement, including dysbiosis, barrier dysfunction, and microbial translocation, has been increasingly recognized in both humans and animal models (25–29). However, the mechanisms linking intestinal alterations and chronic inflammation during VL remain unclear. Given the non-redundant properties of IL-1α and IL-1β (1–3) and the emerging relevance of the gut in VL pathogenesis (25–29), we hypothesized that IL-1 signaling contributes to intestinal barrier maintenance during *L. infantum* infection. In this study, we addressed the roles of IL-1R1, IL-1α, and IL-1β in experimental VL, focusing on the interplay between mucosal integrity, microbiota composition, and inflammatory immunity.

In this study, we reveal a role for IL-1α in promoting the maintenance of the intestinal barrier during murine VL and regulating gamma interferon (IFNγ) production. The absence of IL-1R strengthens the IFNγ response, and IL-1α mediates this effect, while IL-1β is dispensable. The infection induced the expression of IL-1α in the intestine, which is important for microbiota composition and gut barrier integrity. The absence of IL-1α led to microbiota dysbiosis, intestinal permeability, and microbiota translocation. The amplified IFN-γ production is dampened by broad-spectrum antibiotic treatment. These findings indicate that IL-1α protects intestinal barrier function during VL and indirectly regulates the IFNγ-mediated response.

## RESULTS

### IL-1R deficiency strengthens IFNγ-mediated response by IL-1α

**Fig 1:**
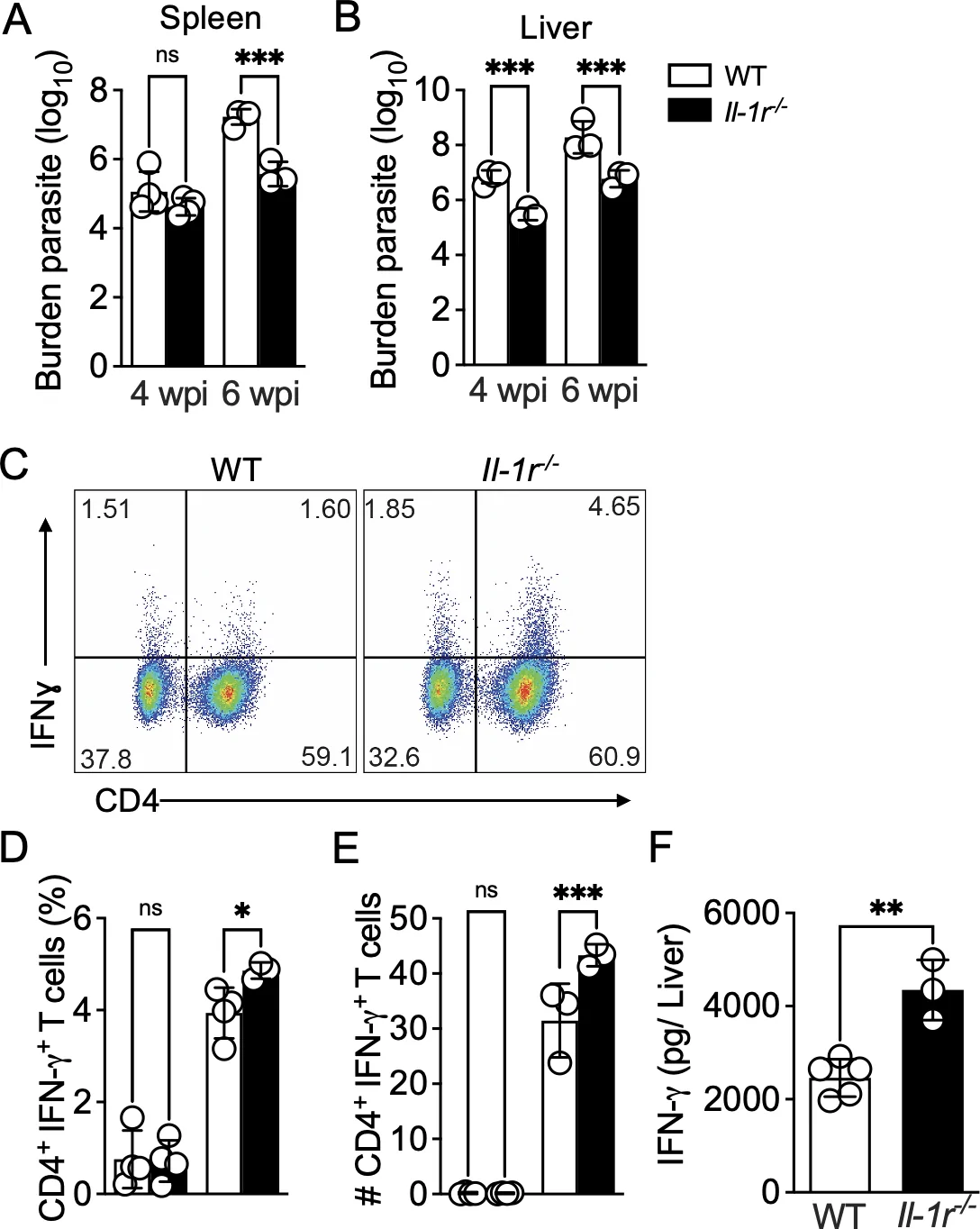
IL-1R strengthens IFNγ-mediated response in the target organs of VL. C57BL/6 WT and *Il-1r^−/−^* mice were i.v. infected with 10^7^ *L. infantum* promastigotes. (**A and B**) Parasite loads were detected in the spleen and liver. (**C**) Representative dot plots of IFNγ production by CD4^+^ T cells from the spleens of *L. infantum*-infected WT and *Il-1r^−/−^* mice in response to polyclonal stimuli. The graph bars represent the (**D**) percentage and (**E**) absolute numbers of IFNγ-producing CD4^+^ T cells in the spleen of naïve (ni) and *L. infantum*-infected WT and *Il-1r^−/−^* mice at 6 weeks post-infection (wpi). (**F**) IFNγ levels in the liver homogenate supernatant. The data are expressed as the means ± SEM and are representative of three independent experiments (*n* = 3 or 4 mice). Statistical significance was calculated by Student’s *t* test (**E**). (*P < 0.05, **P < 0.01, and ***P < 0.001; ns, not significant).

To determine the role of IL-1R, C57BL/6 background of mice knocked out for IL-1R (*Il-1r*^−/−^) and C67BL/6-wild-type (WT) mice were infected with *L. infantum* parasites. Mice were euthanized at 6 weeks post-infection (wpi) for parasite quantification in the target organs. *Il-1r^−/−^* mice harbored significantly lower parasite numbers in the spleen (Fig. 1A) and liver (Fig. 1B) than WT mice. Parasitic control was associated with a higher percentage and number of polyclonally activated CD4^+^ T cells producing IFNγ in the spleen (Fig. 1C through E). In addition, the livers of infected *Il-1r^−/−^* mice contained significantly higher levels of IFNγ than those of WT controls (Fig. 1F).

**Fig 2:**
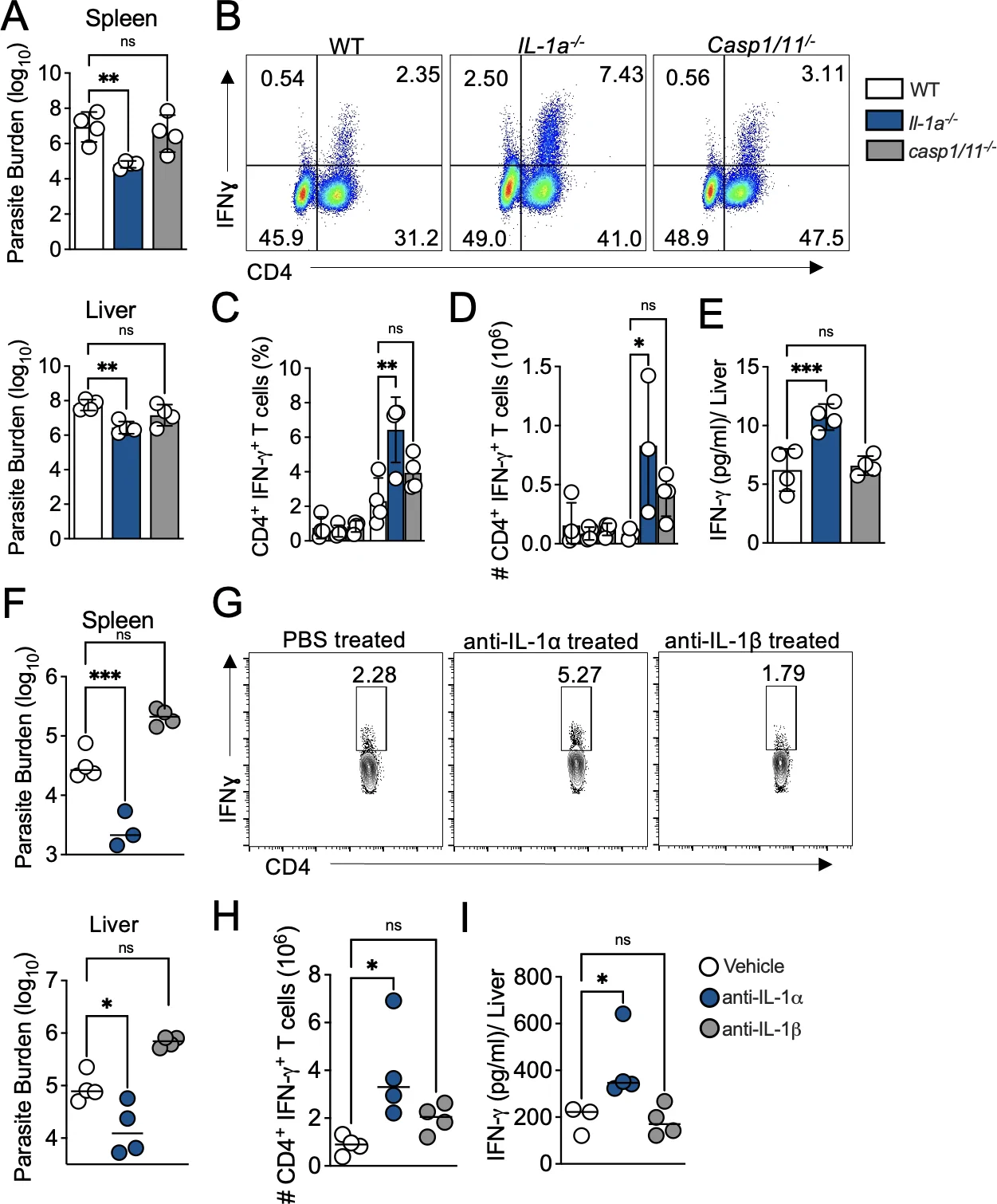
IL-1α, not IL-1β, deficiency strengthens the IFNγ response in the target organs of VL. (**A–E**) WT, *Il-1α^−/−^*, and *Casp1/11^−/−^* mice were infected with 10^7^ *L. infantum* promastigotes. (**A**) Parasite load in the spleen and liver. (**B**) Representative dot plots of IFNγ production by CD4^+^ T cells from the spleens of *L. infantum*-infected WT, *Il-1α^−/−^*, and *Casp1/11^−/−^* mice in response to polyclonal restimulation. Graph bars represent the (**C**) percentage and (**D**) absolute numbers of IFNγ-producing CD4^+^ T cells in the spleen of naïve and *L. infantum*-infected WT, *Il-1α^−/−^,* and *Casp1/11^−/−^* mice. (**E**) IFNγ levels in the liver homogenate supernatant. (**F–I**) C57BL/6 WT mice were i.p. treated with anti-IL-1α or anti-IL-1β monoclonal antibodies or with PBS and i.v. infected with 10^7^ *L. infantum* parasites. (**F**) Parasite loads in the spleen and liver at 6 wpi. (**G**) Representative dot plots of IFN-γ production by CD4^+^ T cells from the spleens of *L. infantum*-infected mice treated with anti-IL-1α, anti-IL-1β, or PBS in response to polyclonal stimuli. (**H**) Graph bars represent the absolute number of CD4^+^ T cells producing IFNγ in the spleens of *L. infantum*-infected mice treated with anti-IL-1α, anti-IL-1β, or PBS. (**I**) IFNγ levels in the liver homogenate supernatant. The data are expressed as the means ± SEM and are representative of three (**A–E**) and two (**F–I**) independent experiments (*n* = 3–5 mice). Statistical significance was calculated by two-way ANOVA (*P < 0.05, **P < 0.01, and ***P < 0.001; ns, not significant).

Furthermore, we infected IL-1α-knockout mice (*Il-1a^−/−^*) or caspase1/11-double knockout mice (*caspase1/11^−/−^*) to evaluate the relative contribution of IL-1α and IL-1β, respectively, during infection. Similar to those of infected *Il-1r^−/−^* mice, the spleen and liver of the *Il-1a^−/−^* mice harbored fewer parasites than the controls (Fig. 2A), and both the frequency (Fig. 2B and C) and the absolute number of IFNγ-producing CD4^+^ T cells were significantly increased (Fig. 2D), following an increase in IFNγ level in the liver compared to the controls (Fig. 2E). No differences in the parasite load in target organs and IFNγ production were found in the *caspase1/11^−/−^* infected mice. In agreement with the phenotype of *Il-1a^−/−^* mice, the treatment with antibody anti-IL-1α, but not anti-IL-1β, reduced the number of parasites in the spleen and liver (Fig. 2F) and significantly increased the number of CD4^+^ T cells producing IFN-γ in the spleen (Fig. 2G and H) and the amounts of IFNγ in the liver (Fig. 2I) compared to the not treated group. Altogether, the results demonstrate that IL-1R and IL-1α negatively regulate the IFNγ-mediated responses in the target organs of VL.

### IL-1α deficiency alters intestinal microbiota composition during *L. infantum* infection

**Fig 3:**
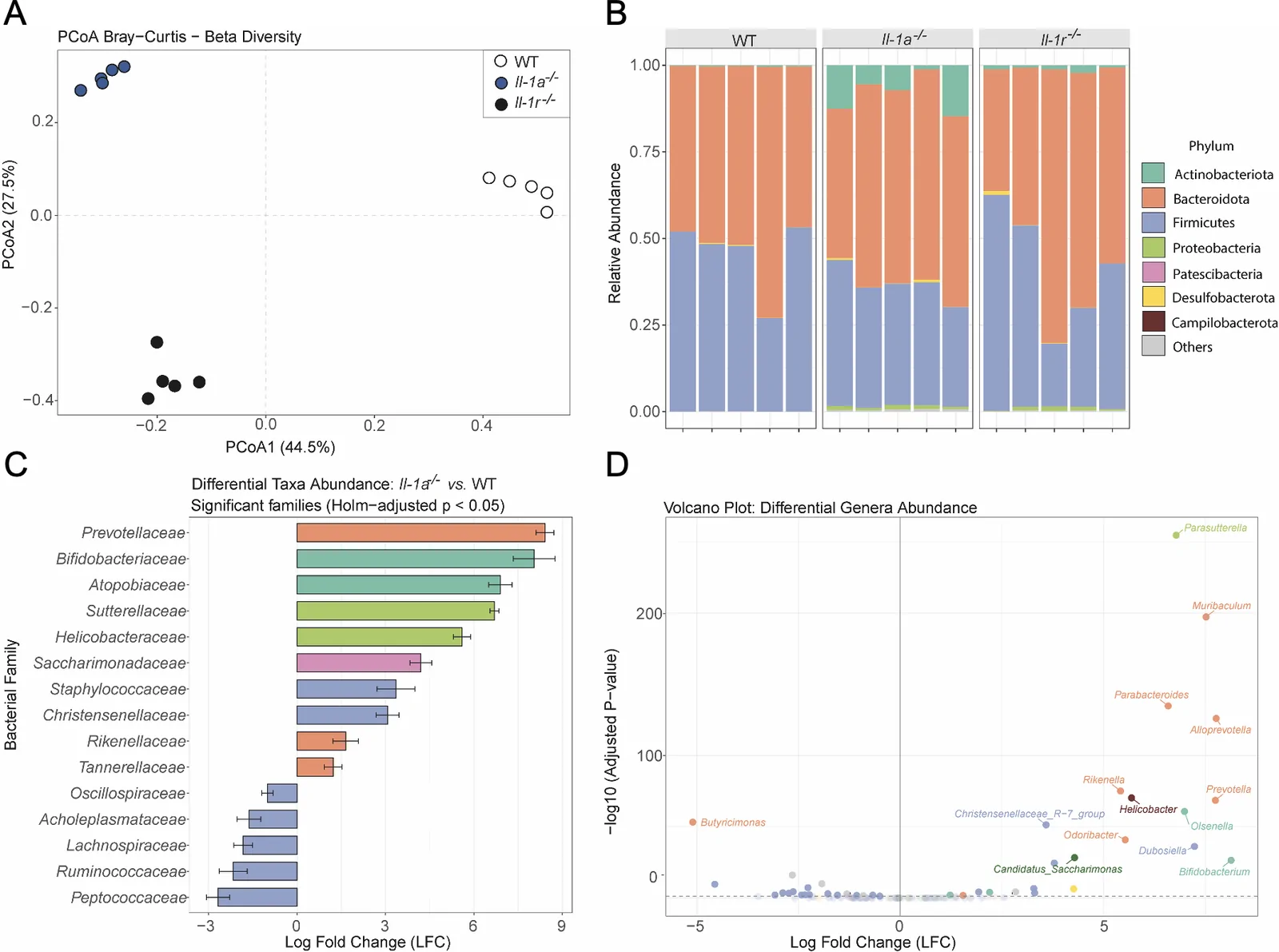
Absence of IL-1α causes intestinal microbiota dysbiosis during VL. Microbiota composition determined by bacterial 16S rRNA sequencing of cecal samples from WT, *Il-1a^−/−^,* and *IL-1r^−/−^* mice (C57BL/6 mice background). (**A**) PCoA based on Bray-Curtis dissimilarities to calculate beta diversity for WT (white), *Il-1a^−/−^* (blue), and *IL-1r^−/^*^−^ (black) mice cecal content microbiota. (**B**) Average proportions of phyla in cecal content of WT, *Il-1a^−/−^,* and *IL-1r^−/−^* mice. (**C**) Grouped bar plot showing the most differentially abundant families in the *Il-1a^−/−^* vs WT-infected mice. LFC represents the variance in proportion between *Il-1a^−/−^* vs WT-infected mice. (**D**) Volcano plot highlighting the overexpressed genus of bacteria present in the *Il-1a^−/−^* vs WT-infected mice.

IL-1α expression affects the composition of intestinal microbiota (30). To test whether IL-1α deficiency directly modifies the gut microbiota of infected mice, we prepared a library of cecal contents of infected *Il-1r*^−/−^, *Il-1a^−/−^*, and WT control mice for 16S rDNA sequencing. The microbiota composition of cecal contents from *Il-1r*^−/−^, *Il-1a^−/−^*, and control mice showed distinct sample clustering based on beta diversity, calculated using Bray-Curtis distances (which quantify differences in microbial community composition based on taxon abundances without incorporating phylogenetic relationships) (Fig. 3A). PCoA analysis revealed clear separation of experimental groups from control along PC1, accounting for 44.63% of the variance. Notably, PC2 (27.44% of variation) further separated *Il-1r*^−/−^ from *Il-1a^−/−^* mice, indicating distinct microbiota compositions between the two knockout genotypes. This separation suggests that IL-1α and IL-1R play different roles in shaping gut microbial communities. Although Bray–Curtis PCoA showed clear clustering of experimental groups, PERMANOVA did not reach statistical significance, likely due to limited sample size and reduced statistical power. Importantly, homogeneity of multivariate dispersions was confirmed (betadisper, ANOVA), indicating that the observed ordination patterns were not driven by differences in within-group dispersion. Among the five dominant bacterial phyla found in the intestinal microbiota, cecal contents from Il-1a^−/−^ showed a reduction in Firmicutes and Bacteroidota, along with an expansion of Actinobacteriota, Proteobacteria, and Patescibacteria compared to WT mice (Fig. 3B). In Il-1r^−/−^ mice, no statistically significant change in Actinobacteriota and Patescibacteria was observed, whereas the other three phyla exhibited the same change pattern (Fig. 3B). Furthermore, two less abundant phyla, Deferribacterota and another unclassified phylum, showed reduction in *Il-1r*^−/−^ mice but not in the *Il-1a^−/−^* model. These differential phylum-level abundances likely account for the separation observed along the PC2 axis in beta diversity PCoA (Fig. 3A). Using ANCOM-BC, differentially abundant (*P*-adjusted value > 0.05; adjust method = “holm”) families were identified. Families enriched in *Il-1a*^−/−^ mice compared to WT mice include Prevotellaceae, Rikenellaceae, and Tannerellaceae (Bacteroidetes phylum), Bifidobacteriaceae and Atopobiaceae (Actinobacteria phylum), Sutterellaceae and Helicobacteraceae (Proteobacteria phylum), Saccharimonadaceae (Patescibacteria phylum), Staphylococcaceae and Christensenellaceae (Firmicutes phylum). Families reduced in *Il-1a*^−/−^ mice compared to WT mice include Oscillospiraceae, Acholeplasmataceae, Lachnospiraceae, Ruminococcaceae, and Peptococcaceae, all belonging to the Firmicutes phylum (Fig. 3C). Using the same analysis, we identified 41 differentially abundant genera in *Il-1a^−/−^* compared to WT mice, of which 21 were enriched. These genera are shown in the volcano plot (unclassified taxa were excluded, Table S1), which highlights a greater number of enriched than depleted taxa. Among the top-enriched genera were Parasutterella, Muribaculum, Parabacteroides, Alloprevotella, Rikenella, Prevotella, Helicobacter, and Olsenella, whereas Butyricimonas showed the strongest depletion (Fig. 3D). These shifts suggest that the loss of IL-1α modulates the intestinal microbiota composition.

### IL-1α is required for intestinal barrier function during *L. infantum* infection

**Fig 4:**
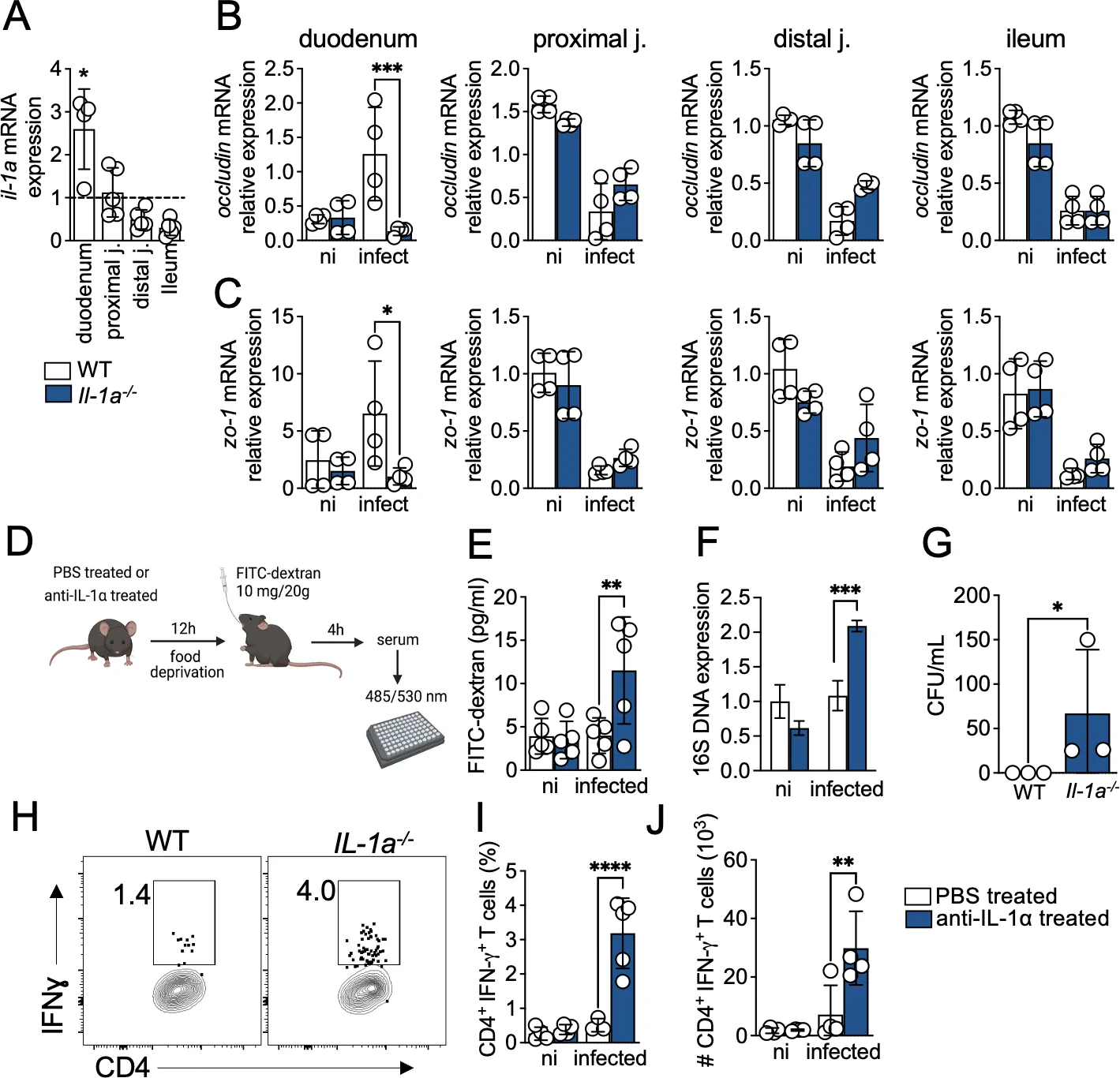
IL-1α is required for intestinal barrier integrity during VL. (**A**) mRNA expression of *Il-1a* in the duodenum, proximal jejunum, distal jejunum, and ileum of C57BL/6 WT-infected mice relative to naïve mice (dashed line). (**B and C**) C57BL/6 WT and *Il-1a^−/−^* mice were infected with 10^7^ *L. infantum* parasites. Expression of mRNA for (**B**) *Occludin* and (**C**) *Zo-1* in the duodenum, proximal jejunum, distal jejunum, and ileum of WT and *Il-1a^−/−^* infected mice relative to WT naïve mice. The data were normalized by the expression of mRNA for β-2-microglobulin. (**D**) C57BL/6 WT mice were i.p. treated with anti-IL-1α monoclonal antibody or with PBS and infected with 10^7^ *L. infantum* parasites. Mice were deprived of food for 12 h and gavaged with FITC-dextran to assess intestinal permeability *in vivo*. (**E**) The concentration of FITC in the serum was assessed 4 h after FITC-dextran administration. (**F**) 16S DNA expression in MLN by qPCR. (**G**) Quantification of culturable aerobic bacteria in the mesenteric lymph nodes of WT and *IL-1a^−/−^* infected mice. Live bacteria were quantified by plating tissue homogenates on BHI agar (*n* = 3 mice). (**H**) Representative dot plots and graph bars of the (**I**) percentage and (**J**) number of CD4^+^ T cells producing IFNɣ in the MLN. The data are expressed as mean ± SEM and represent two independent experiments (*n* = 3–5 mice). The statistical significance was calculated by one-way ANOVA (*P < 0.05, **P < 0.01, and ***P < 0.001).

Intestinal alterations are associated with VL fatal progression in the hamster model and severity in humans (25–29). However, the protective mechanisms involved in intestinal barrier or susceptibility during murine VL still need to be clarified. IL-1 cytokines are implicated in intestinal inflammation and repair processes (17, 30). We hypothesized that the heightened IFNγ inflammation observed in infected *Il-1a^−/−^* mice could result from intestinal barrier dysfunction. We first determined whether IL-1α is induced during infection by quantifying *Il-1a* mRNA expression in the intestine. We evaluated different sections of the intestine (duodenum, proximal jejunum, distal jejunum, and ileum) and observed an upregulation of *Il-1a* mRNA in the duodenum of infected mice compared to that of noninfected mice (dashed line) (Fig. 4A). To further explore the contribution of IL-1α to the intestine during VL, we assessed the expression of major components of tight junction assembly and function, occludin, and ZO-1 (31), by analyzing mRNA in the intestine of infected *Il-1a^−/−^* or WT mice. The transcripts for occludin (Fig. 4B) and ZO-1 (Fig. 4C) were downregulated in the duodenum of *Il-1a^−/−^* infected mice compared to WT mice. No differences in the expression of the tight junction were observed in other sections of the intestine.

Furthermore, we tested intestinal permeability to determine whether the alteration in tight junction expression in the absence of IL-1α also had a functional impact on barrier function. For this purpose, C57BL/6 mice were infected with *L. infantum* and treated with anti-IL-1α antibody or PBS as a control. At 6 wpi, mice received fluorescein isothiocyanate (FITC)-labeled dextran by oral gavage, and subsequent FITC levels were measured in the serum, as shown in the schematic figure (Fig. 4D). Our data show that the infection by itself has no impact on the permeability by comparing infected mice vs non-infected mice. A substantial enhancement of FITC concentration in the serum of infected mice treated with anti-IL-1α compared to the control group (PBS-treated), indicating that intestinal permeability occurs in the absence of IL-1α (Fig. 4E). Extending our above hypothesis, we evaluated the presence of bacteria by 16S in the mesenteric lymph node (MLN) by PCR. There was no detection of 16S DNA in the MLN of WT mice. However, *Il-1a^−/−^* infected mice presented a significant increase in the expression of 16S DNA compared to WT-infected mice (Fig. 4F). Corroborating the data, we found culturable bacteria from MLN of infected *Il-1a^−/−^* mice (Fig. 4G). Consistent with the splenic data (Fig. 1), infected *Il-1a^−/−^* mice presented a pronounced number of CD4^+^ T cells producing IFNγ compared to WT-infected mice in the MLN (Fig. 4H through J). Despite intestinal alterations observed in the absence of IL-1α, no parasites were detected in any of the analyzed sections of the intestine by qPCR or histopathology evaluation (data not shown). The results demonstrate that IL-1α is induced during VL and is required for intestinal barrier integrity.

To evaluate whether the exacerbated IFNγ-mediated response in the absence of IL-1α is *Leishmania*-specific, CFSE-splenic cells from labeled phosphoenolpyruvate carboxykinase (PEPCK)-specific T cell receptor (TCR)-transgenic (CD45.2) mice were transferred to CD45.1 recipient mice, followed by *L. infantum* infection and treatment with an anti-IL-1α antibody or PBS as a control (Fig. S1A). To evaluate the proliferation and IFNγ-production of PEPCK-specific CD4^+^ T cells, flow cytometry analysis was performed on CD45.2^+^ cells and I-Ab PEPCK_335-351_ tetramer^+^ cells (gated on CD4^+^ cells) (Fig. S1B). We observed comparable frequencies of CFSE-labeled cells and IFNγ^+^ cells in both groups of mice treated with anti-IL-1α and the control in the spleen (Fig. S1C and D) and MLN (Fig. S1E and F), while CD4^+^ CD45.1^+^ cells from mice treated with anti-IL-1α have more CD4^+^ cells producing IFNγ (Fig. S1G). These data show that the exacerbated IFNγ-response in the absence of IL-1α is not *Leishmania*-PEPCK-specific.

### Antibiotic cocktail treatment dampens exacerbated Th1 response in the infected *IL-1α^−/−^* mice

**Fig 5:**
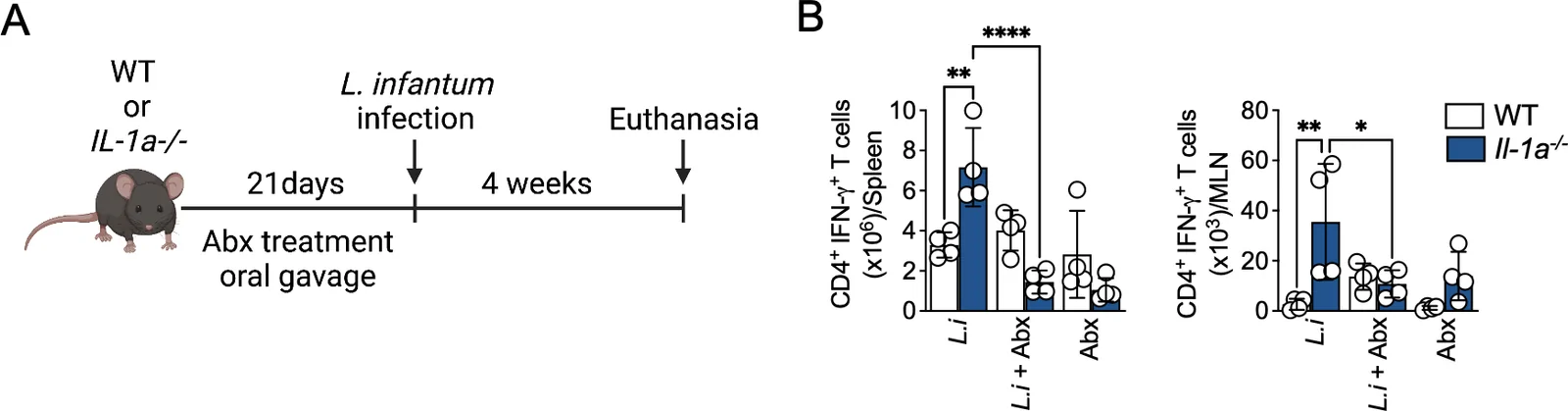
Exacerbated IFNγ-response in *Il-1a^−/−^* infected mice is reversed by intestinal microbiota depletion. (**A**) WT and *Il-1a^−/−^* mice were administered daily doses of a cocktail of antibiotics (Abx) (ampicillin, vancomycin, neomycin sulfate, and metronidazole) for 21 d by oral gavage before *L. infantum* infection. At 4 wpi, the mice were euthanized. (**B**) Representative dot plots and graph bars of CD4^+^ T cells producing IFNɣ in the spleen and MLN of *L. infantum*-infected, *L. infantum*-infected, and Abx-treated or only Abx-treated mice. The data are expressed as the mean ± SEM and represent two independent experiments (*n* = 4 mice). The statistical significance was calculated by one-way ANOVA (*P < 0.05, **P < 0.01, and ***P < 0.001, ****P < 0.0001).

We hypothesized that the microbiota translocation may be contributing to the exacerbation of the IFNγ production in the absence of IL-1α. To explore this possibility, WT or *Il-1a^−/−^* mice were treated daily with a cocktail of antibiotics (metronidazole, vancomycin, ampicillin, and neomycin) (Abx) for 21 days by oral gavage and then infected with *L. infantum* parasites (Fig. 5A), as shown in the schematic figure. After 4 wpi, mice were euthanized and IFNγ production was evaluated in both the spleen and MLN. As demonstrated in Fig. 5B, infected *Il-1a^−/−^* mice presented an increased number of IFNγ-producing CD4^+^ T cells in the spleen and MLN compared to infected WT mice. We observed a significant reduction in CD4^+^ T cells producing IFNγ in the *Il-1a^−/−^* infected mice treated with Abx. These data suggest that the increased IFNγ production is microbiota-dependent and that IL-1α negatively regulates the IFNγ-response in an indirect way.

### Hematopoietic cells are the source of IL-1α in VL

**Fig 6:**
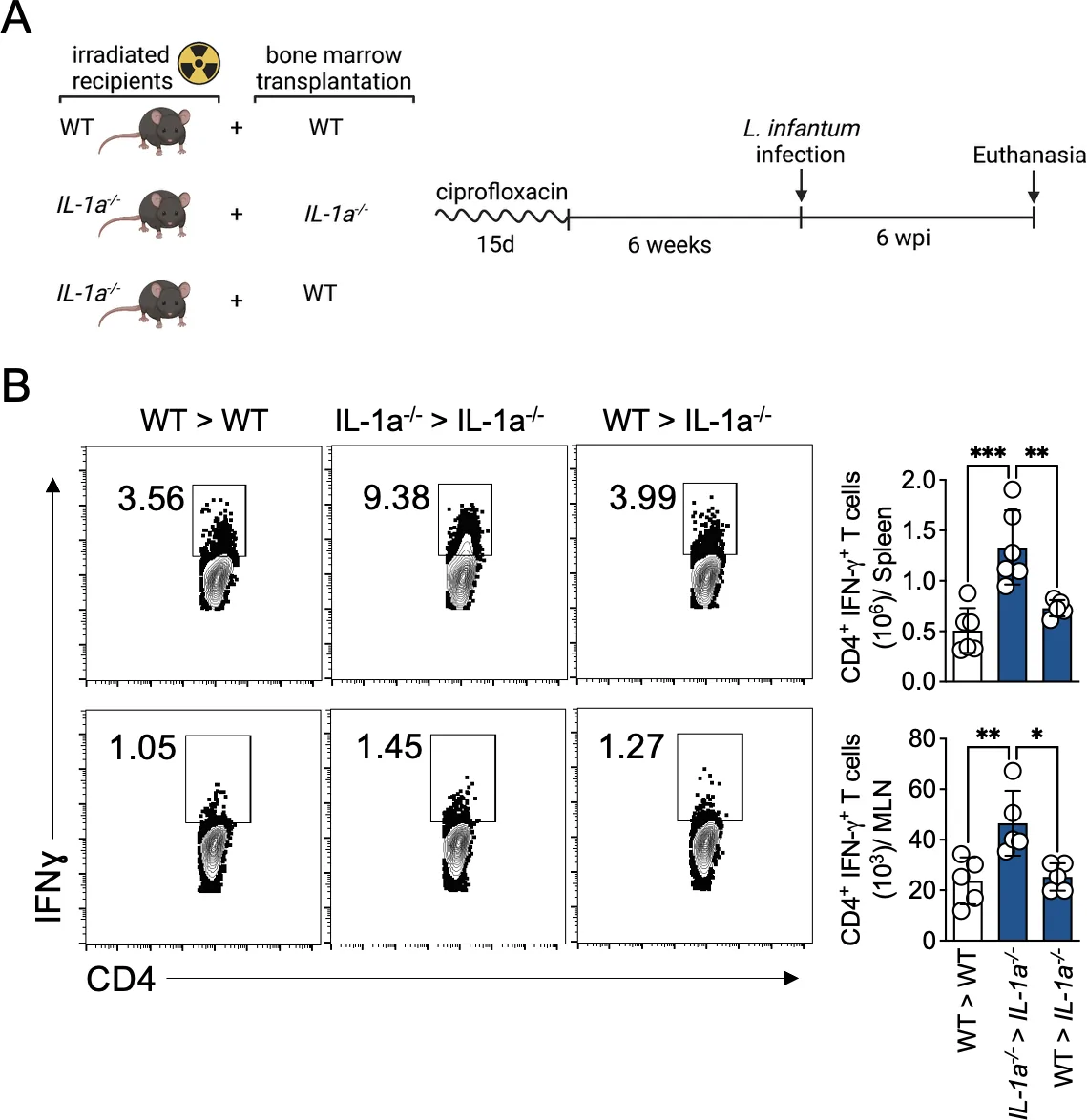
Hematopoietic cells are the source of IL-1α. (**A**) WT and *IL-1a^−/−^* mice were irradiated and repopulated with bone marrow cells from WT or *IL-1a^−/−^* mice. One day after bone marrow (BM) transplantation, mice were treated for 15 days with the antibiotic ciprofloxacin hydrochloride diluted in drinking water. After 6 weeks required for BM engraftment, mice were infected with *L. infantum* and (**B**) euthanized at 6 wpi to assess IFNɣ production by CD4^+^ T cells in the spleen and MLN. The data are expressed as mean ± SEM (*n* = 5 mice). The statistical significance was calculated by one-way ANOVA. (*P < 0.05, **P < 0.01, and ***P < 0.001).

To assess whether hematopoietic cells are the source of IL-1α during VL, we generated bone marrow chimera mice by irradiating *Il-1a^−/−^* recipient mice and repopulating them with bone marrow from WT or *Il-1a^−/−^* mice (WT > *Il-1a^−/−^*). As controls, *Il-1a^−/^*− and WT mice were irradiated and repopulated with WT and *Il-1a^−/−^* bone marrow cells, respectively (Fig. 6A). Reconstitution of irradiated *Il-1a^−/−^* recipients with *Il-1a^−/−^* bone marrow (*Il-1a^−/−^* > *Il-1a^−/−^*) recapitulated the increased Th1 response observed in intact *Il-1a^−/−^* animals. Reconstitution of irradiated *Il-1a^−/−^* with WT bone marrow (WT > *Il-1a^−/−^*) reverted the exacerbated production of IFNγ by CD4^+^ T cells in the spleen and MLN compared to the *Il-1a^−/−^* > *Il-1a^−/−^* group, as shown in Fig. 6B, suggesting that hematopoietic cells are the source of IL-1α during VL.

## DISCUSSION

The intestinal involvement in VL outcome may be underestimated because the diagnosis requires multiple random biopsies, even in the absence of gastrointestinal symptoms, due to the potential asymptomatic parasitism. Here, we demonstrated the role of IL-1α in promoting intestinal homeostasis during murine VL. The infection induced the expression of IL-1α in the intestine, which is important for microbiota composition and gut barrier integrity. The absence of IL-1α led to microbiota dysbiosis, intestinal permeability, and microbiota translocation. The absence of IL-1R/IL-1α strengthens the IFNγ response, and the broad-spectrum antibiotic treatment dampened the exacerbated IFNγ response. These findings reveal a mechanistic link between intestinal homeostasis and inflammation in VL.

Since IL-1α and IL-1β bind and activate the same receptor, they would be expected to present the same biological functions. We have demonstrated that the absence of IL-1R or IL-1α exacerbates the IFNγ-mediated response, not IL-1β. Different from VL, IL-1β has a well-established role in cutaneous leishmaniasis (CL), promoting macrophage activation (19) while also contributing to pathology in specific contexts (20–22, 32). Here, we found no differences in parasite number and IFNγ production in the absence of IL-1β in VL. However, we do not discard the role of IL-1β in modulating innate cell functions at earlier time points of infection, as it has been described to mediate the recruitment of inflammatory cells (11).

Our data using PEPCK-specific T-cell receptor (TCR) transgenic mice suggested that the increased IFNγ-response in the absence of IL-1α is not *Leishmania*-specific. Consistent with these data, broad-spectrum antibiotic treatment reduced IFNγ production in the absence of IL-1α. These data suggest that the increased IFNγ production is a consequence of microbiota translocation. IL-1α recombinant treatment modulates granuloma formation during experimental VL without impact on parasite control (24). Consistent with this, our data suggest that IL-1α does not directly modulate anti-*Leishmania* immunity. Instead, it functions as a regulator of intestinal barrier stability, preventing the spread of microbial products that potentiate inflammation. Infected IL-1R or IL-1α knockout mice harbored fewer parasites in the spleen and liver as a consequence of the increased IFNγ production. Still, possible immunopathological effects might occur since persistent high levels of IFNγ are not associated with parasite control in human VL (33–35). Recent studies have shown that whole blood cells from VL patients produce IFN-γ in response to antigenic activation and identified CD4^+^ T cells as the main type of IFN-γ-producing cells. The levels of IFN-γ were similar before and after successful treatment, suggesting that the inability of these patients to control the disease was not due to a defect in Th1 responses, whereas IL-10 is considered a major marker of disease progression (36, 37). We have previously demonstrated that exacerbated IFNγ inflammation causes liver damage and TLR4-IRF1-IFNβ limits its production (38). Likewise, our data suggest that IL-1α contributes to “disease tolerance,” limiting systemic inflammation.

Intestinal involvement in VL is increasingly recognized, with diarrhea being a prevalent symptom and a consistent risk factor for death (39). Endotoxemia resulting from microbial translocation from the gut is also discussed as a clinicopathological feature (28, 39). Intestinal disorders are common in immunosuppressed and HIV-co-infected patients (25, 40) and are occasionally reported in immunocompetent individuals (27, 41). Hamsters are symptomatic for intestinal VL, exhibiting intestinal parasitism, compromised barrier function, and bacterial translocation, while the murine model is considered resistant to intestinal symptoms (28, 29). On the other hand, infection in mice induces a mild lymphocytic infiltration in the lamina propria and rarely in the submucosa of the duodenum, without detectable parasites (29). Here, we demonstrate that IL-1α contributes to intestinal homeostasis during murine VL. The absence of IL-1α led to microbiota dysbiosis, intestinal barrier permeabilization, and bacterial translocation. We suggest that IL-1α plays a protective role in the maintenance of intestinal function during murine visceral leishmaniasis, and this effect indirectly regulates the IFNγ production. Although we did not directly investigate IL-1α expression or function in hamsters, our findings raise the possibility that species-specific differences in IL-1α regulation may contribute to the distinct outcomes observed in murine and hamster models of visceral leishmaniasis. In mice, infection induces intestinal IL-1α expression, which appears to play a protective role in maintaining gut barrier integrity and limiting excessive systemic inflammation. We speculate that alterations in this regulatory pathway in hamsters could contribute to increased intestinal permeability, enhanced systemic inflammation, and ultimately disease progression. Further studies will be required to determine whether differences in IL-1α production, signaling, or tissue-specific functions underlie the increased susceptibility of hamsters to fatal disease. Our data open a new perspective to identify the factors that contribute to resistance or susceptibility related to intestinal VL symptoms. Further studies regarding the consequences of the altered intestinal barrier function will provide a deeper understanding of the impact of the intestinal environment and consequent systemic inflammation.

The intestinal microbiota emerged as a central component of this process. ABX treatment *IL-1a^−/−^* mice suggest that the increased IFNγ production is microbiota-dependent and that IL-1α negatively regulates the IFNγ response in an indirect way. While our findings support a role for microbiota-dependent signals in driving the enhanced immune response, we cannot exclude the possibility that some of the observed effects may also be influenced by the direct immunomodulatory properties of the antibiotics (42). The absence of IL-1α resulted in a marked shift in microbiota composition, with reduced Firmicutes and expanded Bacteroidota, Actinobacteriota, and Proteobacteria, a pattern that mirrors findings in dogs with clinical VL (43). In humans, higher levels of circulating lipopolysaccharide have been linked to the VL severity (41). Nonetheless, a systematic investigation of the human microbiome in leishmaniasis patients has not yet been undertaken. In mice, preliminary observations suggest that *L. infantum* infection promotes no shifts in the microbial community (data not shown), whereas infected *IL-1a^−/−^* mice differ completely from infected WT mice. Infected *IL-1a^−/−^* mice show a reduction in Firmicutes and an expansion of Bacteroidota, Actinobacteriota, and Proteobacteria compared with WT mice. Our data recapitulate some hallmarks of the microbiota composition of dogs with clinical visceral leishmaniasis, such as contraction in Firmicutes and expansion in the Proteobacteria phylum, and expansion in the Prevotellaceae family (43). The Firmicutes phylum contains a high proportion of shifts in short-chain fatty acids (SCFA)-producing capabilities. The imbalance in the Firmicutes/Bacteroidota ratio is considered a potential biomarker for metabolic health, with changes in its composition linked to shifts in SCFAs profiles and gut barrier integrity (44). Prevotellaceae is linked to chronic inflammatory diseases (45). For example, HIV-induced Prevotella expansion leads to dysfunctional immune responses in the gut mucosa driving bacterial translocation, endotoxemia, and systemic inflammation (46, 47). An expansion of Proteobacteria is a well-documented microbial signature of gut dysbiosis, consistently observed across various gastrointestinal and systemic diseases. The bloom of Proteobacteria is also linked to increased intestinal permeability, bacterial translocation, and systemic inflammation due to exposure to bacterial endotoxins like lipopolysaccharide (47–49). Overall, our microbiome data suggest that IL-1α stabilizes the intestinal ecosystem during infection. More studies are needed to understand the impact of IL-1α on mucosal immune response modulation during VL. As microbiota composition alterations are complex and context-dependent, more investigations should consider factors beyond *Leishmania* infection that can influence the microbiota, such as nutritional status and mucosal immune mechanisms involved in the gut barrier protection.

**Fig 7:**
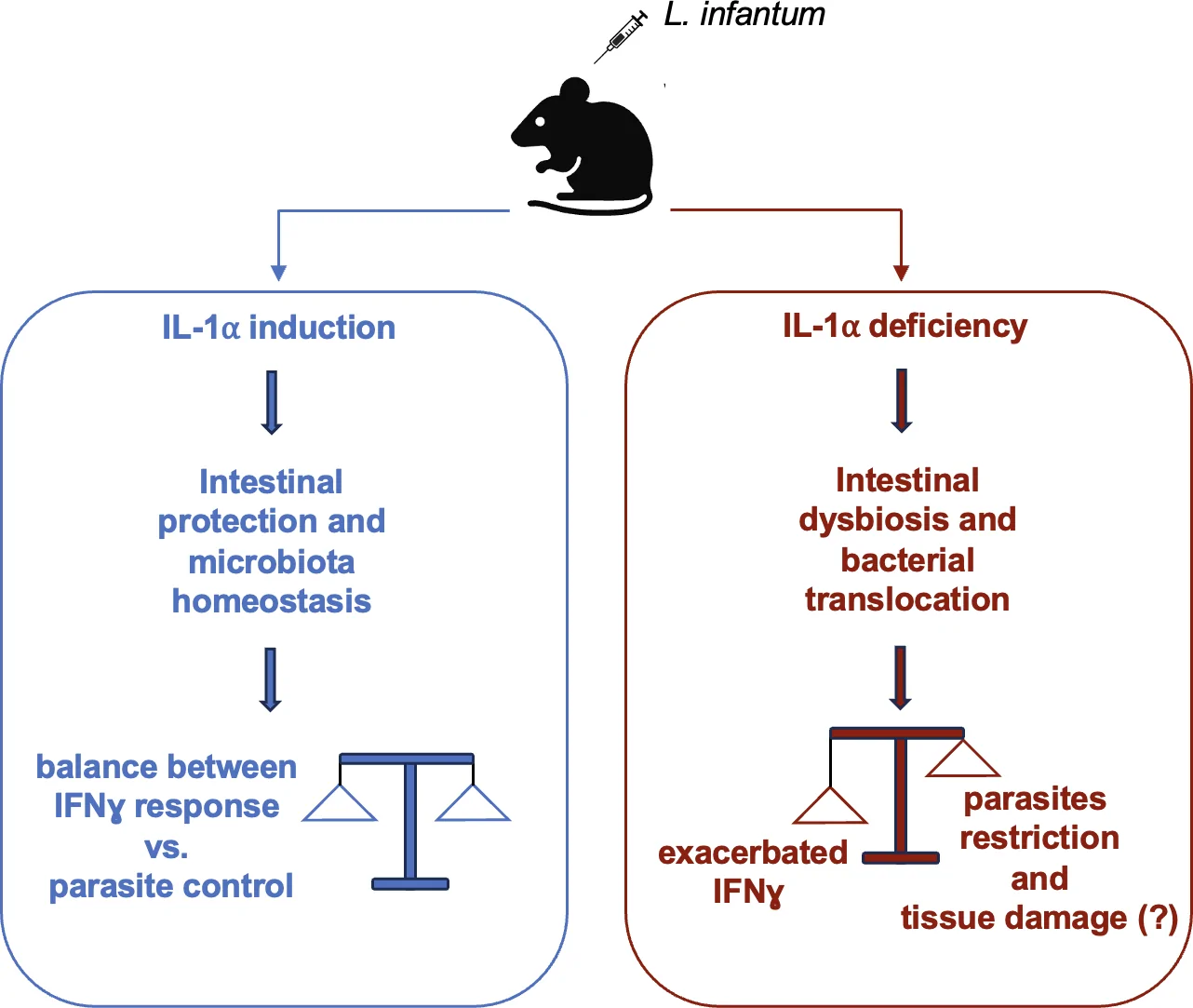
IL-1α protects the intestinal barrier in murine visceral leishmaniasis. *L. infantum* infection induces IL-1α expression in the intestine, which contributes to intestinal integrity by promoting tight junction expression and microbiota homeostasis, avoiding intestinal permeability and bacterial translocation, thereby preventing the systemic spread of microbial products that potentiate inflammation. The absence of IL-1α leads to dysbiosis of the microbiota, increased intestinal permeability, and translocation of the microbiota, resulting in exacerbated IFNγ-mediated inflammation.

IL-1α expression is induced by a wide variety of damage- and pathogen-associated molecular patterns (7) and has been associated with exacerbation of the inflammatory process (4). In this regard, the precursor form of IL-1α released from damaged intestinal epithelial cells following the onset of colitis acts as an alarmin initiating local inflammation. In accordance, anti-IL-1α treatment significantly protected mice from developing acute DSS-induced colitis (30). However, IL-1α can exert nonredundant roles due to its different compartmentalization within the producing cell and the microenvironment. Almost all cells in the intestinal environment express IL-1R and can respond to IL-1α stimulation, and the nature of the insult determines the IL-1R activation in distinct cell types. For example, DSS acts as a toxin directly on colonic epithelial cells, triggering IL-1R on mesenchymal cells. The infection with *Citrobacter rodentium* triggers IL-1R in mesenchymal cells and innate lymphocyte cells, which contribute to epithelial repair (17). IL-1 signaling orchestrates the epithelial regeneration response of the skin, lung, and intestine (15–17). Corroborating this concept, we have demonstrated that IL-1α contributes to intestinal barrier integrity. In agreement with our findings, IL-1α plays a protective role in gut-derived sepsis induced by *Pseudomonas aeruginosa* colonization (50). The exogenous administration of IL-1α ameliorates intestinal ischemia and reperfusion injury by reducing intestinal permeability and bacterial translocation during endotoxemia (51). Also, IL-1α/IL-1R signaling can be triggered by living cells under stress or infection without necrotic cell death (52). Our study extends these observations to VL, demonstrating that IL-1α is induced in the duodenum by the infection, even though parasites are not detected in the intestine of mice (28, 29). This suggests that systemic inflammatory cues or low-grade mucosal activation are sufficient to trigger IL-1α-dependent protective responses. Therapeutic modulation of IL-1α represents a promising strategy for managing visceral leishmaniasis. IL-1α targeting could mitigate systemic pathology by preserving gut epithelial integrity and indirectly dampening the exacerbated IFN-γ response. However, its potent pro-inflammatory nature requires a finely tuned clinical approach. Taken together, our findings position IL-1α as a key mediator linking intestinal barrier integrity to systemic immune regulation in VL. The infection induces IL-1α expression in the intestine, which contributes to intestinal integrity by promoting tight junction expression and microbiota homeostasis, thereby avoiding intestinal permeability and bacterial translocation (Fig. 7). The present study expands the knowledge of intestinal dysfunction in the outcome of VL.

## MATERIALS AND METHODS

### Animals

Six- to eight-week-old mice were used for the infection experiments. Female C57BL/6 (wild type [WT]), C57BL/6 *Il-1r^−/−^*, C57BL/6 *Il-1a^−/−^*, and C57BL/6 *Casp1/11^−/−^* mice were obtained from the animal facilities at the School of Medicine of Ribeirão Preto, University of São Paulo (Brazil). The mice were housed in temperature-controlled rooms (22–25°C) at the animal facility of the Department of Biochemistry and Immunology (School of Medicine of Ribeirão Preto) and received water and food *ad libitum*.

### Parasite culture, infection, and load estimation

*L. infantum* (isolate HU-UFS14) was cultured in Schneider medium with 20% heat-inactivated fetal bovine serum (FBS), 5% penicillin and streptomycin (both from Sigma‒Aldrich, St. Louis, MO, USA), and 2% male human urine. Parasitic virulence was maintained by serial passaging in BALB/c mice and by culturing *in vitro* for no more than five passages. Mice were intravenously infected via the retro-orbital plexus with 10^7^ stationary-phase *L. infantum* promastigotes in 100 μL of phosphate-buffered saline (PBS). A quantitative limiting dilution assay determined the hepatic and splenic parasite burdens.

### *In vivo* α-IL-1α and α-IL-1β antibody treatment

C57BL/6 mice were treated intraperitoneally with 500 μg/mouse α-mouse IL-1α antibody (ALF-161 and BE0246, respectively) (BioXCell, West Lebanon, NH, USA) in PBS twice a week until 6 wpi. A corresponding IgG isotype antibody or PBS served as a control (Miltenyi Biotec).

### Bone marrow cell isolation and generation of chimeric mice

WT and *Il-1a^−/−^* bone marrow (BM) stem cells were obtained by femur flushing. The bone was surgically removed, and muscle tissue was scraped away from the bone using a scissor and scalpel blade. Subsequently, the epiphysis was cut, and the stem cells were removed from the diaphysis by flushing with RPMI 1640 medium using a syringe with a 26-gauge needle. To generate the chimeras, 10- to 12-week-old WT and *Il-1a^−/−^* mice were irradiated using a cesium-137 source irradiator (Mark I model 25) at 9 Gy. One day after irradiation, 5 × 10^6^/200 µL BM cells were intravenously injected. The mice were divided into the following groups: WT mice repopulated with WT BM cells, *Il-1a^−/−^* mice repopulated with *Il-1a^−/−^* BM cells, and *Il-1a^−/−^* mice repopulated with WT BM cells. After BM transplantation, the mice were treated for 15 days with the antibiotic ciprofloxacin hydrochloride diluted in drinking water at 10 mg/mL per mouse. After 2 months (the period required for BM engraftment), the mice were intravenously infected via the retro-orbital plexus with *L. infantum* parasites.

### PEPCK-cell transference

To evaluate the parasite-specific immune response, splenic cells from phosphoenolpyruvate carboxykinase (PEPCK)-specific T-cell receptor (TCR)-transgenic mice were CFSE labeled, and 8 × 10^6^/100 µL were intravenously injected into CD45.1 recipient mice, followed by *L. infantum* infection. Mice were treated twice a week with anti-IL-1α antibody or PBS as a control, as described above. Flow cytometric analysis of *Leishmania*-specific CD4^+^ T cells was performed based on live cells, CD4^+^ cells, CD45.2^+^ cells, and I-A^b^–PEPCK_335–351_ tetramer^+^ cells.

### FITC–dextran uptake assay

To directly assess intestinal permeability *in vivo*, mice were gavaged with 10 mg/20 g of 4 kDa FITC–dextran (Sigma‒Aldrich) per mouse at a volume of 100 mL after food deprivation for 12 h. Four hours postinoculation, mice were killed, and serum was collected and measured for FITC concentration using a plate reader (Tecan, Maennedorf, Switzerland). FITC was measured against a standard curve of serially diluted FITC–dextran, and the plate was read with an excitation wavelength of 485 nm and an emission wavelength of 528 nm.

### Antibiotic treatment

Mice were administered daily doses of 1.86 mg/mouse ampicillin (Sigma‒Aldrich), 0.96 mg/mouse vancomycin (Sigma‒Aldrich), 1.86 mg/mouse neomycin sulfate (Sigma‒Aldrich), and 1.86 mg/mouse metronidazole (Sigma‒Aldrich) diluted in 300 μL of drinking water by oral gavage for 21 days before *L. infantum* infection.

### Antibodies and flow cytometry

Antibodies against the following mouse proteins were obtained from BioLegend: CD3 (145-2C11), CD4 (RM4-5), IFNγ (XMG1.2), CD45.1 (A20), CD45.2 (104), and IL-1α (ALF-161). For leukocyte identification, cells from the spleen and MLN were first gated based on their characteristic size (FSC) and granularity (SSC); doublet cells and dead cells were excluded; cells were gated on CD45^+^ cells; and T lymphocytes (CD3^+^CD4^+^ cells) were identified. The total leukocyte counts were determined by measuring the relative expression of the leukocyte subpopulations stained with a specific antibody within a set of 300,000 acquired events as a proportion of the leukocyte number obtained in a Neubauer chamber. For intracellular staining, single-cell suspensions were aseptically prepared from spleen and MLN, diluted to a concentration of 2 × 10^6^ cells/mL, and seeded into 24-well plates in a total volume of 500 μL of complete RPMI 1640 medium (1 × 10^6^ cells/well; Gibco). The cells were cultured with PMA (50 ng/mL) and ionomycin (500 ng/mL; Sigma‒Aldrich, St. Louis, MO, USA) as well as brefeldin A (BioLegend, San Diego, CA, USA) for 4 h. The cells were then fixed with 4% paraformaldehyde and permeabilized with 0.5% saponin. Cell acquisition was performed using a LSR Fortessa flow cytometer. The data were plotted and analyzed using FlowJo software (TreeStar, Ashland, OR, USA). The antibodies used are listed in the Supporting Protocol.

### Quantitative real-time PCR

Total RNA was isolated from the intestine using the SV Total RNA Isolation System Kit (Promega, Madison, WI, USA), and complementary DNA (cDNA) was generated using Transcriptase Reverse SuperScript III (InvivoGen). SYBR Green Mix-based real-time qPCR assays were performed using the StepOnePlus Real-Time PCR System (Applied Biosystems). The gene expression levels were normalized to the glyceraldehyde-3-phosphate dehydrogenase (GAPDH) expression level.

### DNA extraction and 16S rRNA gene PCR analysis

DNA extraction from the MLNs was performed using a DNeasy Blood and Tissue kit (QIAGEN) following the manufacturer’s instructions. For the PCR analysis, 10 ng of DNA and 5 μM of the 16S primer (forward, 5′-TGG CTC AGG ACG AAC GCT GGC GGC-3′; reverse, 5′-CCT ACT GCT GCC TCC CGT AGG AGT-3′) were used. The amplification was performed in a QuantStudio 12K Flex instrument (Applied Biosystems).

### Microbiota analysis

Cecal contents were collected from WT, *Il-1a^−/−^*, and *IL-1r^−/−^* infected mice for 16S rRNA sequencing and analysis. DNA was extracted from cecal contents with the PowerSoil DNA Isolation kit (Qiagen) according to the manufacturer’s protocol for 16S rDNA sequencing and analysis. Amplicon library preparation and sequencing were performed by ByMyCell using an Illumina platform (paired-end, 2 × 250). The V3–V4 hypervariable regions of the bacterial 16S rRNA gene were amplified using the following primers: 341F (5′-CCTAYGGGRBGCASCAG-3′) and 806R (5′-GGACTACNNGGGTATCTAAT-3′). Sequencing data were delivered demultiplexed, with non-biological nucleotides removed. Downstream analysis was performed using DADA2 pipeline (53). Only forward reads were used due to a lack of significant overlap between forward and reverse reads, and the higher quality scores of forward reads compared to reverse reads. Processed data were imported into the phyloseq package (54), and diversity analysis was performed in rarefied data. Differential abundance analysis was conducted using ANCOM-BC (55) with default settings. Plots were generated using R packages. All scripts and details of the R libraries used are publicly available on GitHub (see “Data availability”).

### Statistical analysis

The analyses were performed using Prism 5.0 software (GraphPad Software, Inc.). Each variable from data collected in the experiments was subjected to analysis of normal distribution and homogeneity of variance. Parametric analyses such as Student’s *t* test and analysis of variance (ANOVA) were applied to data presenting normal distribution and variance homogeneity. The means obtained for the different groups were compared by one-way ANOVA followed by the Bonferroni *post hoc* test. Comparisons between two sample groups were performed using unpaired two-tailed Student’s *t*-test. The data are expressed as the means ± SEM and are representative of two to four independent experiments. Differences were considered significant at *P < 0.05, **P < 0.01, and ***P < 0.001.

## Data Availability

The complete 16S rRNA sequencing and data used to perform the analysis presented in this article are available in the GitHub repository at https://github.com/gabcmartins/IL1a-IL1R_microbiota_analysis.

## SUPPLEMENTAL MATERIAL

Fig. S1 mbio.00657-26-s0001.tif IFNγ response of PEPCK-specific cells is not affected by IL-1⍺ neutralization.

Supplemental file mbio.00657-26-s0002.docx Supplemental legends.

Table S1 mbio.00657-26-s0003.xlsx Results of the ANCOM-BC differential abundance analysis.

ASM does not own the copyrights to Supplemental Material that may be linked to, or accessed through, an article. The authors have granted ASM a non-exclusive, world-wide license to publish the Supplemental Material files. Please contact the corresponding author directly for reuse.
