# Supplementary material for: IL-1α protects intestinal barrier in murine visceral leishmaniasis
Source: mBio. 2026 Sep 14;17(10):e00657-26. doi: 10.1128/mbio.00657-26 (PMC13712978; doi:10.1128/mbio.00657-26)
Supplement: Supplementary file 1 [file mbio.00657-26-s0002.docx]

**S1 Fig. IFNƔ-response of PEPCK-specific cells is not affected by IL-1⍺ neutralization.** (A) Spleen cells from donor PEPCK-specific (CD45.2) mice were CFSE-labeled and transferred to CD45.1 recipient mice. CD45.1 mice were subsequently infected with *L. infantum* parasites and treated with anti-IL-1$\alpha$ antibody or PBS. (B) Flow analysis strategy of *Leishmania*-specific CD4^+^ T-cells was performed based on live cells, CD4^+^ cells, CD45.2^+^ cells, and I-A^b^–PEPCK_335–351_ tetramer^+^ cells. (C and F) Representative dot plots and graph bars of CFSE^+^ cells and IFN-ɣ production by CD45.2^+^ cells from the spleen (C and D) and MLN (E and F) of *L. infantum*-infected mice treated with anti-IL-1α or PBS. (G) Graph bar of IFN-ɣ production by CD45.1^+^ cells from the spleen and MLN. The data are expressed as the mean ± SEM (n = 4 mice). The statistical significance was calculated by using Student’s t-test.

**Supplemental Table 1. Results of the ANCOM-BC differential abundance analysis comparing *Il-1a*⁻/⁻ and WT mice.** Adjusted p-values (q values) < 0.05 (Holm correction) were considered differentially abundant. The table includes log fold change (LFC), standard error (SE), p-value, adjusted p-value (q-value), and differential abundance status (diff_abn). Only phylum, family, and genera classified as differentially abundant (diff_abn = TRUE) are shown. Unclassified taxa and features assigned to other taxonomic levels were excluded from the volcano plot.
